# Unraveling Plant Cell Death during *Phytophthora* Infection

**DOI:** 10.3390/microorganisms10061139

**Published:** 2022-05-31

**Authors:** Kayla A. Midgley, Noëlani van den Berg, Velushka Swart

**Affiliations:** Department of Biochemistry, Genetics and Microbiology, Hans Merensky Chair in Avocado Research, Forestry and Agricultural Biotechnology Institute, University of Pretoria, Pretoria 0002, South Africa; kayla.midgley@fabi.up.ac.za (K.A.M.); noelani.vdberg@fabi.up.ac.za (N.v.d.B.)

**Keywords:** plant pathology, necrosis, hemi-biotroph, agroinfiltration, CRN

## Abstract

Oomycetes form a distinct phylogenetic lineage of fungus-like eukaryotic microorganisms, of which several hundred organisms are considered among the most devastating plant pathogens—especially members of the genus *Phytophthora*. *Phytophthora* spp. have a large repertoire of effectors that aid in eliciting a susceptible response in host plants. What is of increasing interest is the involvement of *Phytophthora* effectors in regulating programed cell death (PCD)—in particular, the hypersensitive response. There have been numerous functional characterization studies, which demonstrate *Phytophthora* effectors either inducing or suppressing host cell death, which may play a crucial role in *Phytophthora’s* ability to regulate their hemi-biotrophic lifestyle. Despite several advances in techniques used to identify and characterize *Phytophthora* effectors, knowledge is still lacking for some important species, including *Phytophthora cinnamomi*. This review discusses what the term PCD means and the gap in knowledge between pathogenic and developmental forms of PCD in plants. We also discuss the role cell death plays in the virulence of *Phytophthora* spp. and the effectors that have so far been identified as playing a role in cell death manipulation. Finally, we touch on the different techniques available to study effector functions, such as cell death induction/suppression.

## 1. Introduction

Pathogens within the oomycete genus *Phytophthora* are among some of the most destructive plant pathogens globally, causing disease and significant losses in important agricultural and forestry crops, damaging the environment, as well as impeding attempts to mitigate climate change [1,2,3,4]. One of the more well-known incidences of *Phytophthora* disease is the Irish Potato Famine in 1845. This incident was caused by *Phytophthora infestans*—the causal agent of late blight of potatoes. The disease resulted in the death of half of the potato crop that year and about three-quarters of the crop over the next seven years [3,5]. Other *Phytophthora* spp., which cause significant impact worldwide, include the causal agents of sudden oak death in California (*Phytophthora ramorum*), stem rot of soybean (*Phytophthora sojae*), black shank of tobacco *Phytophthora nicotianae*), phytophthora root rot of avocado and jarrah dieback of trees in the Jarrah Forest, both caused by *Phytophthora cinnamomi* [3,6,7]. Despite the economic and ecological relevance of *P. cinnamomi*, the mechanisms this pathogen utilizes to infect and successfully colonize its hosts are still largely unknown. *P. cinnamomi* is known to infect plants that are important for agriculture and forestry, with the most significant food losses occurring in avocados. There is little to no knowledge on how *P. cinnamomi,* a hemi-biotrophic pathogen, maintains a biotrophic lifestyle early in the infection and a necrotrophic lifestyle later in the infection.

Pathogenic lifestyles are centered around feeding on host tissue, where success is dependent on the pathogen’s ability to overcome host defenses. One host defense strategy *Phytophthora* spp. must evade to sustain their biotrophic phase is the hypersensitive response (HR). The HR is a form of programed cell death (PCD) and is generally the last resort in a host plant’s defense response against a pathogen. The response involves the localized death of cells surrounding the initial site of infection to inhibit the spread of the pathogen. Later in the infection, the HR is favored during the necrotrophic phase. Studies have shown that *Phytophthora* spp. manipulate the host plant’s cell death machinery to elicit a susceptible outcome [8,9]. 

*Phytophthora* spp. harbor a distinct set of genes involved in moderating host–pathogen interactions [10]. These genes encode effectors—small, secreted proteins—that interfere with host defense processes. There are two groups of effectors, cytoplasmic and apoplastic effectors, which are classified by where in the host cell they act. The most well-studied classes of *Phytophthora* cytoplasmic effectors are Crinklers (CRNs) and RxLRs (Arg-x-Leu-Arg, where x is any amino acid) [4]. Research into *Phytophthora* effectors has greatly expanded due to the availability of genomic and transcriptomic data, allowing for the prediction of putative effector homologs in *Phytophthora* spp. [11]. These valuable tools—followed by functional characterization techniques, such as transient transformation in model plants—allow for the identification of effectors that may play crucial roles during infection. However, little genomic research has been conducted on *P. cinnamomi*, which leaves a gap in knowledge on the mechanisms employed by this pathogen to successfully infect and cause disease in economically and ecologically important plants.

Due to their economic impact, *Phytophthora* spp. are some of the most studied among oomycetes [4,12]. There is, however, still limited knowledge on the mechanisms utilized to regulate cell death in host plants. It is likely these processes are determined by the delivery of functionally distinct pathogen effectors into the host cell [13]. In this review, the role of *Phytophthora* effectors in host cell death induction and suppression is discussed by reporting on forms of cell death, recent studies of *Phytophthora* effectors involved in host cell death and technological advances, which have aided in the identification and characterization of effectors.

## 2. Programed Cell Death in Host Plants

Plants are immobile organisms and have had to develop morphological, biochemical and physiological adaptations to survive in their environment. PCD is an important mechanism for plant development or defense and can be triggered by both abiotic and biotic stressors [9,14,15]. PCD is described as a genetically controlled process where selected cells are eliminated through a coordinated multi-step fashion [15]. This phenomenon is of considerable importance in agriculture because PCD can significantly affect plant health and subsequent yield [16,17]. Therefore, it is important to understand both the triggers and the pathways through which PCD is elucidated.

### 2.1. Classification of PCD

**Figure 1:**
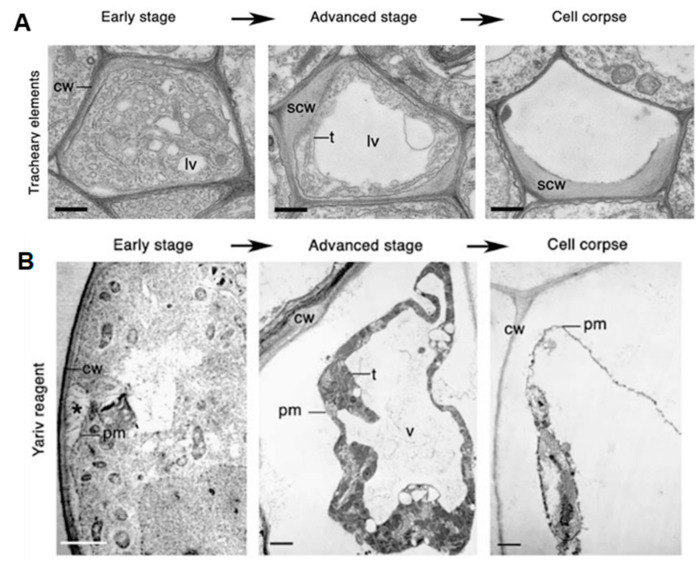
Classes of cell death. (**A**) Vacuolar cell death. Electron micrographs of programed cell death (PCD) in *Arabidopsis* tracheary elements. cw, cell wall; lv, lytic vacuole; n, nucleus; scw, secondary cell wall; t, tonoplast. Scale bars, 500 nm (tracheary elements). Manifests by a gradual decrease in cytoplasm volume and an increase in lytic vacuole volume. (**B**) Necrotic cell death. Electron micrographs of Yariv-reagent-induced death in the *Arabidopsis* cell culture. Asterisks denote the detachment of plasma membrane form the cell wall during early stages of cell death. c, chloroplast; cw, cell wall; pm, plasma membrane; t, tonoplast; v, vacuole. Scale bars, 2 µm. There is an absence of a growing lytic vacuole, and there is early rupture of the plasma membrane, which results in shrinkage of the protoplast. Pictures of *Arabidopsis* treachery elements were republished with authors’ permission from Avci, U.; Petzold, E.; Ismail, I.O.; Beers, E.P.; Haigler, C.H. Cysteine proteases XCP1 and XCP2 aid micro-autolysis within the intact central vacuole during xylogenesis in *Arabidopsis* roots. Plant J. 2008, 56, 303–315, https://doi.org/10.1111/j.1365-313X.2008.03592.x [21] and those of the Yariv-reagent-induced cell death were republished with authors’ permission from Gao, M.; Showalter, A.M.; Yariv reagent treatment induces PCD in *Arabidopsis* cell cultures and implicates arabinogalactan protein involvement. Plant J. 1999, 19, 321–331, https://doi.org/10.1046/j.1365-313X.1999.00544.x [22].

There has been some confusion regarding how different forms of PCD should be classified and how terminology should be standardized. Cell death is classified based on the morphological characteristics and, as a result, two major classes of PCD are proposed to occur in plant biology [14]. Class one is vacuolar cell death, which involves the engulfment of the cytoplasm by lytic vacuoles, uptake and degradation of portions of the cytoplasm in the vacuolar lumen and, finally, rupture of the tonoplast followed by a massive release of vacuolar hydrolases. This results in the rapid destruction of the entire protoplast—a cell whose cell wall has been removed by enzymes—or, in some cases, even the entire cell, including the cell wall (Figure 1A). Class two is necrotic cell death, which is distinguished from vacuolar cell death by mitochondrial swelling, absence of the growing lytic vacuoles and early rupture of the plasma membrane, resulting in shrinkage of the protoplast (Figure 1B). Necrosis is regarded as an acute death response, which develops rapidly, taking anywhere from several minutes to a day to complete [14]. The use of morphology to classify PCD has allowed a better understanding of how cell death manifests. Although one limitation in this is that a well-known form of PCD, known as the HR, cannot be ascribed to either class, as its development displays characteristics of both vacuolar and necrotic cell death [14,18,19,20].

The HR is a special form of PCD, involving rapid localized cell death at the point of pathogen penetration [16,23]. The host plant utilizes HR to limit biotrophic pathogen growth and generates long-range signals for systemic acquired resistance (SAR) [24]. Thus, another PCD classification system was developed to accommodate the placement of the HR. This system classifies forms of PCD based on what functions they play in the host plant, rather than by their morphology or pathways. Two classes were described: developmentally controlled PCD (dPCD) and pathogen-triggered PCD (pPCD). During vegetative and reproductive development, dPCD occurs and is often a final differentiation step for specific cell types [25]. Conversely, pPCD is elicited in the host plant by invading agents and can benefit either the plant or pathogen, depending on the studied plant–pathogen interaction [26]. An additional class has also been proposed to describe PCD resulting from environmental stress, termed ePCD [25]. The ePCD classification includes stresses, such as temperature or irradiation, or biotic aggressors, such as pathogens [15]. pPCD is specific to pathogen-triggered cell death, whereas ePCD includes all external stressors as PCD triggers. The use of ePCD as a classification may, however, be problematic, since different PCD pathways may be in play during both abiotic and biotic-triggered PCD.

### 2.2. Programed Cell Death in Host Plants

Plant PCD pathways are not as well understood as animal cell death. Animals have a core PCD machinery that is mainly regulated post-translationally [17,27], whereas it is not known whether the different forms of plant PCD share the same core machinery or whether the similarities they share were independently adopted to fulfill analogous roles for different pathways [9]. When looking at the two main plant PCD forms—dPCD and pPCD—there are marked differences as well as commonalities in their proposed pathways. For one, a vacuolar type of cell death is associated with dPCD, and features of both necrosis and vacuolar PCD are seen in pPCD [9]. There is also evidence of transcriptional regulation and signaling in both forms of plant PCD, but in different contexts. Unfortunately, there are still gaps in our knowledge regarding pPCD. This is largely due to predominance of previous dPCD-centered investigations. In addition, variability has been seen in pPCD responses to a multitude of different abiotic and biotic factors, whereas dPCD is a relatively conserved process across all plant species. This section serves to summarize our current knowledge on the transcriptional regulation, hormonal signaling and triggers involved in pPCD.

#### 2.2.1. Transcriptional Regulation of pPCD

The stimulation and repression of cell death pathways by transcription regulators has been seen in animal PCD [28,29], and recent evidence indicates that some level of transcriptional control of PCD is also likely in plants [30,31,32,33,34]. Different classes of transcription factors (TFs), including members of NAC, ethylene-responsive element-binding factors (ERFs) and WRKY families, have been shown to play roles in cell fate regulation in response to different stresses. NAC TFs have been linked to the regulation of PCD triggered by both abiotic and biotic stresses [34]. One example is that of OsNAC4, which has been shown to be a key positive regulator of the HR by modulating the expression of almost 150 genes in rice, such as Copper Zinc Superoxide Dismutase 1 (*CSD1*) gene and BAX Inhibitor 1(*BI-1*) gene [35]. ERF TFs also play a role in the regulation of the HR, where the conditional expression of NbCD1—from *Nicotiana benthamiana*—in response to multiple HR elicitors is sufficient to induce the HR [30]. Numerous WRKY TFs are involved in the regulation of cell death, and they may play a role in the suppression of the HR during initial infection of the necrotrophic fungus, *Botrytis cinerea*, in *Arabidopsis* [36], through the activation or suppression of antagonistic signaling pathways, such as salicylic acid (SA), ethylene (ET) and jasmonic acid (JA) mediated pathways. Although there is a large body of research on TFs and their role in pPCD, there is still a lack of knowledge on *Phytophthora* pathogens and the involvement of TFs in eliciting or suppressing PCD during *Phytophthora* infection.

#### 2.2.2. Phytohormone Signaling Pathways Involved in pPCD

Different phytohormones play a role in dPCD, such as JA, auxin, strigolactones and ET—ET being the most characterized dPCD hormone [37,38,39]. Phytohormones control the dPCD processes via transcriptional regulation of genes, such as proteases and nucleases, to gradually build up dPCD competence during cellular differentiation. This contrasts with pPCD, where no preparation is required, and the cells are always ready to initiate an immune response upon pathogen attack [9]. The infection strategy of a plant pathogen—whether the pathogen adopts a biotrophic, necrotrophic or hemi-biotrophic lifestyle—determines the underlying mechanism for phytohormone-regulated pPCD in the host during plant–pathogen interactions [40,41]. It has been shown that SA plays an essential role in host defense response against biotrophic and the early stages of hemi-biotrophic pathogens, whereas JA and ET play an important role in the host defense response against necrotrophic and the later stages of hemi-biotrophic pathogens [41]. SA is the only phytohormone shown to play an essential role in the establishment of pPCD, allowing immunity toward biotrophic pathogens and susceptibility to necrotrophic pathogens. [42,43]. It has been found that some pathogens interfere with cellular SA biosynthesis or signaling through the delivery of effector proteins. [26]. For example, penetration-specific effector 1 (PSE1) from *Phytophthora parasitica* inhibits SA-mediated cell death and increased pathogen growth by promoting auxin accumulation at infection sites [44]. Due to the importance of phytohormones in the different trophic interactions, it would be of value to investigate their roles in the maintenance and switch from the biotrophic to necrotrophic stage in hemi-biotrophic pathogens, such as *Phytophthora*. This will shed light on how *Phytophthora* is able to successfully infect a host plant and avoid the hosts’ defense responses.

#### 2.2.3. Triggers of pPCD

dPCD requires preparation before PCD can be triggered/executed. Several cytoplasmic signals are implicated in dPCD triggering, such as calcium fluxes, accumulation of reactive oxygen species (ROS) and cytoplasmic acidification [45]. During the self-incompatibility (SI) response—the inability of a plant with functional pollen to set seeds when self-pollinated—in *Papaver rhoeas*, calcium influx triggers a signaling cascade, which results in rapid PCD of the incompatible pollen tubes [46]. In contrast, pPCD requires no preparation and is only triggered upon pathogen attack. The main pPCD trigger is cytoplasmic immune receptor-mediated recognition at the site of attack [47]. Calcium influxes, as well as accumulation of SA, ROS and nitric oxides (NO), are triggered upon pathogen perception during pPCD. SA signaling subsequently amplifies the ROS burst in a positive feedback loop, creating a toxic environment [48]. Some necrotrophic pathogens have been known to ‘hijack’ PCD machinery, where pathogens, such as *Cochliobolus victoriae,* secrete PCD triggering toxins [49,50]. Common triggers that are recognized by host receptors are effectors. Different *Phytophthora* effectors and their role in host PCD will be discussed in a later section.

## 3. Cell Death and *Phytophthora* Virulence

Different forms of pPCD will benefit either the plant or pathogen, depending on the type of plant–pathogen interaction and the trophic lifestyle of the pathogen [4,9,25]. Most *Phytophthora* spp. are hemi-biotrophic pathogens, meaning they feature a biotrophic life stage during early infection followed by a switch to necrotrophy during the later stages of host tissue colonization [4,51]. As the HR is generally considered most effective against biotrophic pathogens, while potentially benefiting necrotrophic pathogens, hemi-biotrophic pathogens—such as *Phytophthora*—are at a distinct advantage [52,53]. This response is a race between the host and pathogen, where the pathogen attempts to tip the balance toward suppression of host defense, and the host tries to launch an effective defense response to prevent infection [4]. 

*Phytophthora* spp. may have developed a strategy to ‘hijack’ a plant’s HR machinery, suppressing the HR during the biotrophic stage and inducing it during the necrotrophic stage [24,54,55]. This ‘hijack’ strategy is further supported by the production of haustoria that deliver defense-controlling pathogenicity factors and effectors, which function in keeping the host cell alive [56,57]. Conversely, the switch to necrotrophy, which involves the upregulation of specialized effector genes—such as Nep1-like proteins (NLPs)—aims to deliberately kill the host cell [58]. This is further supported by the similarities in metabolic enzyme expression between *P. infestans* during the necrotrophic stage and the necrotrophic pathogen *Pythium ultimum* [59]. This strategy increases the virulence of *Phytophthora* spp. through the differential expression and delivery of effectors at different stages of host plant infection and colonization [13,54,55,60,61,62].

## 4. *Phytophthora* Effectors That Induce or Suppress Cell Death

*Phytophthora* has a large repertoire of effector proteins that serve different functions during infection. These effectors produce metabolic or structural changes in host cells, aiding in the growth of the pathogen and disease development [63]. Effectors can be divided into two main groups, namely apoplastic and cytoplastic effectors. Cytoplastic effectors are translocated to the cell cytoplasm where they interact with host targets. Apoplastic effectors are secreted into the extracellular space between cells and interact with targets within the extracellular space, as well as on the host cell surface [4,57,63,64]. 

Cell death plays an important role in plant–pathogen interactions, which has driven a long-standing interest in the characterization of effectors that are able to induce/suppress plant cell death [65]. Genomic resources and methods for characterizing effector functions have allowed for a better understanding of their evolution and role in disease progression. Our understanding of the effector repertoire and their roles in infection in *Phytophthora* spp. with a broad host range, such as *P. cinnamomi*, remains limited [66].

### 4.1. Apoplastic Effectors

Apoplastic effectors are known to act on host targets outside of the host plant cells or on plant cell surface receptors. There has been significant progress in the identification of apoplastic effectors, which induce cell death in host plants. To date, 61 cell-death-inducing apoplastic proteins have been identified in 15 *Phytophthora* spp. (Table 1). A number of these proteins belong to the pectate lyase (PL), glycoside hydrolase (GH) and PcF toxin families. The majority of the apoplastic effectors identified are, however, elicitins and Nep1-like protein (NLPs), which will be discussed in further detail below.

#### 4.1.1. Elicitins

**Figure 2:**
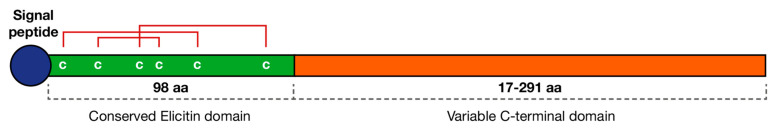
Structure of a *Phytophthora* elicitin. The conserved elicitin domain generally consists of 98 amino acids and contains 6 cysteine residues at conserved positions that form three disulphide bridges. The variable C-terminal tends to be rich in threonine, serine and proline residues.

Elicitins are a conserved class of apoplastic proteins produced by oomycetes—in particular *Phytophthora* and some *Pythium* spp. [121,122]. Elicitins are involved in binding to sterols, which is believed to serve an essential role in *Phytophthora* development and pathogenicity [123,124]. The majority of elicitins possess a signal peptide, a highly conserved 98-amino-acid domain (Pfam PF00964), and a C-terminal domain of variable length (17–291), which is usually rich in threonine, serine and proline residues (Figure 2) [122,125]. Elicitins may elicit a cell death response by their recognition as microbe-associated molecular patterns (MAMPs), resulting in triggering the HR rather than a specific necrotizing activity of the protein itself [126,127].

It was originally believed that elicitins induced cell death via the disruption of plasma membrane integrity upon sterol binding, but *Phytophthora* mutants producing elicitins unable to bind to plant sterols still elicited cell death responses [123,128]. Studies have shown that the elicitin-induced HR involves a ROS burst. It has been proposed that mitogen-activated protein kinases (MAPKs) phosphorylate WRKY7/8/9/11 TFs, resulting in a sustained ROS burst that leads to cell death upon elicitin perception [129]. Oomycete plant pathogens, such as *Phytophthora,* are believed to have evolved an effector toolbox to modulate host responses triggered by their elicitins [126]. This is evident when considering Avr3a-KI from *P. infestans,* which suppresses the HR triggered by INF1 [130]. Additional screens have also revealed that over 30 effectors from different oomycete species suppress INF1-triggered responses [126].

#### 4.1.2. Nep1-like Protein (NLPs)

**Figure 3:**
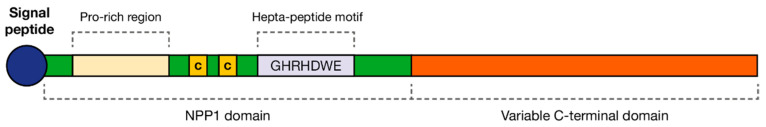
Structure of *Phytophthora* cNLPs. A signal peptide is present followed by a necrosis-inducing *Phytophthora* protein 1 (NPP1) domain containing a 30–45 proline rich region and a Hepta-peptide GHRHDWE motif at around 110–130 aa. In cNLPs, there are two conserved cysteines present between the Pro-rich region and Hepta-peptide motif—ncNLPs have four conserved cysteines in this region.

NLPs are apoplastic effector proteins, which contain an N-terminal secretion signal peptide and a common necrosis-inducing *Phytophthora* protein 1 (NPP1) domain (Figure 3) [65]. This family of effector proteins has been found in bacteria, fungi and oomycete plant pathogens—with the genus *Phytophthora* possessing the largest NLP gene family, which is highly conserved among species [58,65,131]. NLP effectors have been shown to induce cell death and elicit strong immune responses in dicotyledonous plants [65,111,132]. NLPs can be separated into two functional classes: cytolytic (cNLPs) and noncytolytic (ncNLPs), where the specific activity of cNLPs is to cause cell death [133,134,135,136].

It has been suggested that cNLPs may play an important role in the transition of *Phytophthora* spp. from the biotrophic to necrotrophic phase [58]. This is evident by the increase in expression of *PsojNIP* and *PiNPP1*—from *P. sojae* and *P. infestans*, respectively—during the infection stages, coinciding with the transition from biotrophy to necrotrophy [105,111]. Further evidence of this role is seen in *P. capsici*, where *NLP2*, *NLP6* and *NLP14* contribute greatly to the induction of necrosis during infection—like that of *PsojNIP* [137]. Contrastingly, there have been studies reporting that ncNLP genes from *P. infestans*, *P. megakarya*, *P. capsici* and *P. cactorum* were expressed during developmental stages and the early biotrophic infection phase [71,131,137,138]. This may suggest that NLPs play additional roles in virulence, but the exact functions have yet to be resolved [4].

A *P. cinnamomi NPP1* has been reported in a study where the authors investigated the expression of the gene using RT-qPCR, both in vitro, using different carbon sources, and in vivo, during infection of *Castanea sativa* roots [139]. A decrease in *NPP1* expression was noted between 12 and 24 h post-inoculation (hpi) with a significant increase at 36 hpi, suggesting a complex host–pathogen interaction. Although this study shed light on the function of this effector during *P. cinnamomi* infection, further research is required to fully understand the mechanisms underlying the defense mechanisms against *P. cinnamomi* necrosis-inducing proteins.

### 4.2. Cytoplasmic Effectors

#### 4.2.1. RxLRs

**Figure 4:**
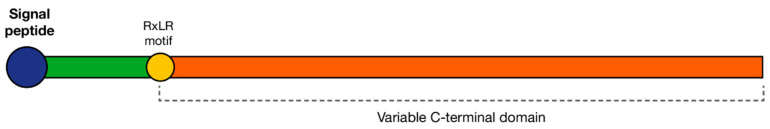
*Phytophthora* RxLR effector structure. Illustration of the characteristic features of *Phytophthora* RxLRs. These effectors have a signal peptide followed by a conserved RxLR (Arg-x-Leu-Arg) motif and a variable C-terminal.

*Phytophthora* RxLRs are cytoplasmic effectors with a modular architecture, including an N-terminal signal peptide for protein secretion, a conserved RxLR motif to facilitate translocation into host cells and a diverse C-terminal domain executing virulence activity (Figure 4) [64,140,141,142]. The RxLR effector family is the largest class of translocated effectors and is specific to *Phytophthora* spp., with there being 560, 370, 390 and 238 RxLR-containing protein coding genes in the genomes of *P. infestans*, *P. ramorum*, *P. sojae* and *P. cinnamomi*, respectively [12,64,143]. These effectors localize to many subcellular organelles and structures, where they target a wide range of pathways throughout the plant cell [4,144]. A key role of RxLRs is the suppression of PAMP-triggered immunity (PTI) and effector-triggered immunity (ETI), where multiple *Phytophthora* RxLRs from different species have been reported to suppress plant cell death triggered by elicitins or other effectors [130,141,145,146].

RxLR effector PsAvh238 from *P. sojae* was found to either induce cell death *in planta* or suppress elicitin-induced plant cell death, depending on the different regions of Avh238 and distinct subcellular localizations [145]. The N-terminal of PsAvh238 and nuclear localization are critical to induce cell death, while the C-terminal and cytoplasmic localization are sufficient for INF1-induced cell death suppression. This illustrates how different localization can convey different RxLR functions. Interestingly, it has also been found that some RxLR effectors may alter the localization of host targets [4,146]. Another example is *P. sojae* RxLR (PsAvh52), which suppresses cell death and defense mechanisms in the early stages of infection by ‘hijacking’ a transacetylase enzyme (GmTAP1) [146]. PsAvh52 relocates GmTAP1 to the cell nucleus where it chemically modifies the host DNA’s packaging, resulting in the activation of nearby susceptibility genes, suppressing the host plant’s defense system.

Some *Phytophthora* RxLRs may also contribute to the establishment of the pathogen’s necrotrophic life stage. This is seen in *P. capsici* RxLR effector PcAvh1, which triggers cell death when expressed in *N. benthamiana*, tomato and bell pepper leaves. This effector is rapidly induced during early infection stages and then exhibits a decline in expression through 3 to 24 hpi but is upregulated again at 36 and 72 hpi [147]. It has previously been proposed that *P. capsici* switches from the biotrophic to necrotrophic lifestyle sometime between 18 and 42 hpi, suggesting that PcAvh1 may help facilitate this switch. PcAvh1 may still play a role during initial infection during the biotrophic stage, but other effectors may inhibit its necrotic activity during early infection. 

There have not been any functional characterization studies performed on suspected *P. cinnamomi* RxLRs. There has, however, been a study reporting to have identified and characterized the *Avr3a* gene from online genomic *P. cinnamomi* sequences by using in silico approaches alone [148]. The authors report that the gene encodes a recognized 209 amino acid protein in the host cytoplasm, where it triggers cell death. However, it should be noted that in silico analysis is not sufficient to definitively assign the function of an effector. It can be used to identify putative effectors; in vivo functional characterization is still required to confirm in silico inferences. Therefore, further in vivo techniques, such as transient transformation via *Agrobacterium,* should be used to confirm this study’s findings.

#### 4.2.2. Crinklers

**Figure 5:**
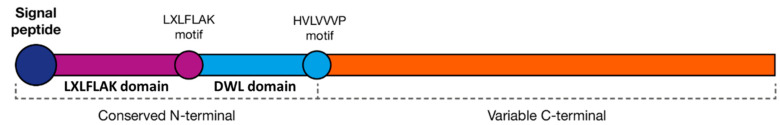
*Phytophthora* CRN effector structure. Diagram illustrating the LXLFLAK and DWL domains, which contains the characteristic motifs within the N-terminal and C-terminal. Featuring two conserved motifs (LXLFLAK and HVLVVP) in the N-terminal, followed by a variable C-terminal. CRNs do not always possess a signal peptide, as there are other secretion pathways.

CRNs are modular proteins that were first identified in *P. infestans* and classified as genes causing crinkling and necrosis [149]. These effector proteins possess a conserved N-terminal containing an LXFLAK, HVLVXXP and DWL motif, which functions in translocation of the CRN proteins from the apoplast into the plant cytoplasm (Figure 5) [150,151]. This is followed by a variable C-terminal, which conveys different functions, including subcellular localization required for the effector function [8,54,60,152]. *Phytophthora* spp. examined thus far have large multigene families of CRN genes, with 196 in *P. infestans*, 100 in *P. sojae* and 49 in both *P. ramorum* and *P. cinnamomi* [11,130]. Unlike RxLR effectors, CRN effectors arose early in oomycete evolution and then later diverged across plant pathogenic species, suggesting CRN effectors play an essential role in oomycete pathogenesis in plants [99,153,154,155,156,157].

Although CRN effectors were first noted to induce crinkling and necrosis in plant tissue, recent studies have shown that the majority of CRNs act in suppressing host cell defenses [131,138,151]. Functional characterization of *Phytophthora* CRNs has provided substantial evidence for the involvement of this class of effectors in the modulation of PCD during infection [24,54,55,60,158]. 

**Figure 6:**
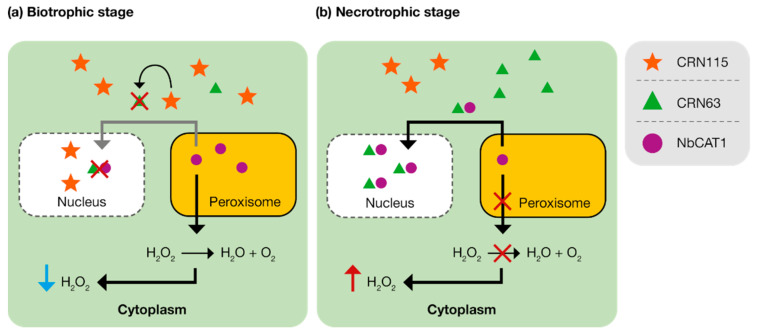
Schematic of how CRN63/115 modulates PCD in *Nicotiana benthamiana*. (**a**) During the early stages of infection (biotrophic stage), CRN115 inhibits the activity of CRN63, preventing the relocation and scavenging of NbCAT1. NbCAT1 is then able to convert H_2_O_2_ into water and oxygen. Inhibiting H_2_O_2_ accumulation induced by CRN63. (**b**) CRN63 is slightly induced during the late stages of infection (necrotrophic stage) and relocates NbCAT1 to the nucleus where NbCAT1 is destabilized and therefore unable to convert H_2_O_2_ into water and oxygen. This results in an accumulation of H_2_O_2_ in the cytoplasm, resulting in PCD.

Some *Phytophthora* spp. have been shown to have at least two CRNs with contradicting functions—where one suppresses cell death, and the other induces cell death—with both required for virulence [26,54,55]. One example is that of CRN63 and CRN115 from *P. sojae*, which induce contrasting and apparently opposite responses when expressed in *N. benthamiana* [24,54]. CRN63 induces cell death, and CRN115 suppresses cell death induced by PsojNIP or CRN63; both CRNs act on catalases to alter H_2_O_2_ accumulation (Figure 6). The stability of catalase proteins is reduced by CRN63, which in turn enhances H_2_O_2_ accumulation and results in the triggering of PCD. Conversely, CRN115 suppresses PCD by inhibiting H_2_O_2_ accumulation induced by CRN63. This mechanism is also employed by *P. parasitica*, where CRN7 and CRN20 function analogously to *P. sojae* CRN63 and CRN115, respectively [54]. These observations are further supported by the differential expression of *CRNs* at different pathogen life stages; for example, *CRN63* shows a 2.8-times-increased expression during late stages of infection [43]. Together, these findings indicate that different CRNs have distinct functions during either the biotrophic or necrotrophic *Phytophthora* spp. life stages.

Unfortunately, there has yet to be any functional characterization studies on *P. cinnamomi* CRNs. Studies such as these are of great interest, since it is suggested that these effectors play an essential role in early infection and regulating PCD [149]. Recently, there have been analyses of expression data for 49 putative *P. cinnamomi CRNs,* and it was found that 11 *CRNs* were significantly expressed, with 1 *CRN* being upregulated compared to mycelia at 120 hpi in avocado, and the remaining 10 demonstrating downregulation at the same time point [11]. This suggests that the majority of *P. cinnamomi* CRNs may function in suppressing the host defenses during the earlier stages of infection, although further expression data at earlier time points and characterization studies are required to definitively conclude the function of *P. cinnamomi* CRNs.

## 5. Techniques Used in the Functional Characterization of *Phytophthora* Effectors

A method commonly used in determining the function of *Phytophthora* effectors is agroinfiltration. Agroinfiltration is an *Agrobacterium tumefaciens*-based method for transient expression of genes of interest *in planta* [66]. This assay is efficient in numerous dicot plant species and is therefore broadly applied in screenings and research in molecular plant–pathogen interactions [159,160,161,162]. Agroinfiltration is also a well-established method to use for the functional characterization of pathogen effectors when that pathogen cannot be regularly transformed, as in the case of *P. cinnamomi*. This approach has been utilized in multiple studies to determine the cell death induction or suppression abilities of *Phytophthora* effectors [8,24,150,151,163]. The use of agroinfiltration coincides with the use of model plants, in particular *N. benthamiana*, which is widely used to study a variety of plant pathogens [164]. This is because *N. benthamiana* expressed sequence tags (ESTs) share similarities with important agricultural Solanaceous crops. Therefore, functional genomics research of host–pathogen interactions conducted in *N. benthamiana* will most likely reveal genes, which play similar roles in agronomically important crops.

The available genome sequences of *Phytophthora* spp. have allowed for a better understanding of the repertoire of effectors utilized by these pathogens, as well as their possible mechanisms to promote pathogen success [10,165]. Genomic and transcriptomic data allow for the prediction of putative effector homologs in *Phytophthora* spp. Tools such as RNA sequencing (RNA-Seq) are useful for gene expression profiling, which aids in identifying pathogenicity genes and predicting what functions they may have. Genome data for *Phytophthora* spp. are accumulating and have been utilized in large-scale transcriptome analyses. This will aid future research to identify key effectors, which may play a role in infection and disease development.

*P. cinnamomi* genomic data have been lacking. This is surprising due to the economic and ecological relevance of *P. cinnamomi* [11]. A recent study has generated a high-quality reference genome for *P. cinnamomi* using a combination of Nanopore and Illumina sequencing platforms, opening up future research on *P. cinnamomi* effectors and their functions [11]. This is an improvement on the five existing, highly fragmented draft genome sequences currently available for *P. cinnamomi* [166,167]. The assembly of the *P. cinnamomi* genome indicated that *P. cinnamomi* has a much larger genome size than what was previously estimated and has allowed better identification and characterization of various pathogenicity-related genes. Therefore, this genome serves as an important foundation for future studies.

Dual RNA-seq has enabled investigations of both host and pathogen transcriptomics simultaneously [156]. This technology allows for the detection of minute amounts of pathogen RNA, and it is more sensitive than either microarrays or northern blotting [168,169,170]. This tool also provides more information, as it provides a picture of global gene expression. RNA-seq data have also allowed for the identification of over 1300 putative pathogenicity genes from cyst and germinating cyst phases of *P. cinnamomi*, of which several encoded for effector proteins that served as candidates for further research [145,171]. An analysis of *P. cinnamomi* dual RNA-seq data from *Eucalyptus nitens*—5 days following inoculation with *P. cinnamomi*—revealed that a putative *P. cinnamomi CRN* effector was highly upregulated, and a pathogenicity-related (*PR-9*) gene was downregulated [156]. This and other evidence in the study demonstrate that a *P. cinnamomi CRN* and a *E. nitens PR-9* gene may play essential roles in causing a susceptible host–pathogen interaction. RNA-seq data have also been used in a separate study to assign possible functions to three *P. cinnamomi* RxLRs [143]. However, further functional characterization is required to conclusively assign functions to any putative effector identified using gene expression data. Research aimed at characterizing effectors from different *Phytophthora* spp. has become essential to understanding the mechanisms these pathogens utilize to invoke a susceptible response in host plants. The transformation of *Phytophthora* spp. has been one method used to deduce the function of effectors; however, some species—such as *P. cinnamomi*—have had limited success in transformation [66,172,173]. The speculated reasons for these limitations are the identification of oomycete promotors and selectable markers to select for transformants [66,172]. Transformation protocols for *Phytophthora* spp., such as *P. capsici*, *P. parasitica* [174] and *P. infestans,* [175] have been successfully produced, but these protocols will not necessarily work for all *Phytophthora* spp.; notably, these protocols have not been successful in *P. cinnamomi* [66]. Although, recently, a proposed protocol has been developed using a PEG/CaCl_2_-mediated protoplast transformation method, where three *P. cinnamomi* transformants were successfully produced in a single isolate [172]. These results have been reproduced in the same *P. cinnamomi* isolate in two separate laboratories (Nanjing Forestry University and Oregon State University). Nonetheless, this protocol still needs to be reproduced in different laboratories and using different strains before it can be validated as a standard protocol for *P. cinnamomi* transformation. 

The development of efficient transformation protocols for various *Phytophthora* spp. will enable future research aimed at characterizing the effectors implicated in PCD. This, in turn, will provide some insight into how different *Phytophthora* spp. are able to maintain the biotrophic and necrotrophic stages during infection in order to achieve a susceptible outcome in host plants. Further work can also be conducted to understand the cell death pathways that may be involved and the host targets, allowing for improved screening for susceptible rootstocks to be used in agricultural practices. 

## 6. Conclusions

PCD in plants is a complicated process with no one single mechanism, and the HR is of particular interest during the plant pathogen–host interaction. The HR can either benefit or be detrimental to host plants, depending on when it is triggered and what infection strategy is employed by the pathogen. There is evidence that *Phytophthora* effectors either directly or indirectly induce/suppress cell death, which ultimately aids in the virulence of the pathogen. This indicates that a possible infection strategy involves the ‘hijacking’ of the HR machinery to benefit the specific life stages of the pathogen. CRNs and NLPs may play a key role during the maintenance of the biotrophic and necrotrophic life stages and therefore require further investigation. Other *Phytophthora* spp., such as *P. cinnamomic*, which are detrimental to numerous economically important agricultural crops, should be investigated to identify effectors that may be involved in regulating host plant cell death. This will entail the development of *P. cinnamomi* transformation approaches, which will allow for better analysis of specific effectors and their functions. Until this can be performed, agroinfiltration serves as an efficient method to study effector proteins’ ability to induce/suppress cell death in *Phytophthora* spp.

## Figures and Tables

**Table 1:** Apoplastic cell-death-inducing proteins identified in *Phytophthora* spp.

Protein Family	Plant Cell Surface Receptor	Co-Receptor	Protein	*Phytophthora* spp.	Function	References
**ND**	-	-	PB90	*Phytophthora boehmeriae*	Induces cell death	[67,68,69]
**Elicitin**	ELR	BAK1, HSP70, HSP90, NbLRK1, SGT1, SRC2-1	Cacto	*Phytophthora cactorum*	Induces cell death	[70]
			PcELL1		Induces cell death	[71]
			PcINF1		Induces cell death	[72]
			Capsicein	*Phytophthora capsici*	Induces cell death and increases defense against *P. nicotianae* in *Nicotiana benthamiana*	[73]
			PcINF1		Induces cell death and pepper defense response	[74,75]
			Cinnamomin	*Phytophthora cinnamomi*	Induces cell death and protects *N. benthamiana* against pathogens	[76,77]
			15-kDa glycoprotein	*Phytophthora colocasiae*	Induces cell death and SAR	[78]
			Cryptogein	*Phytophthora cryptogea*	Induces cell death, SAR and defense of *N. benthamiana* against *P. nicotianae*	[73,79,80,81,82,83,84,85]
			Dreα, Dreβ	*Phytophthora drechsleri*	Induces cell death	[86]
			Hibernalin1	*Phytophthora hibernalis*	Induces cell death	[87]
			INF1	*Phytophthora infestans*	Triggers HR dependent on HSP70, HSP90 and SGT1	[88,89,90,91,92,93]
			INF2A, INF2B		INF2A-induced necrosis dependent on SGT1	[92]
			MgMα, MgMβ	*Phytophthora megasperma*	Induces cell death	[94]
			α-megaspermin, β-megaspermin, γ-megaspermin/32 kDa glycoprotein		Induces cell death, PR gene expression and SAR	[95,96]
			Palmivorein	*Phytophthora palmivora*	Induces cell death	[97]
			Parasiticein/parA1/elicitin 310/elicitin 172	*Phytophthora parasitica*	Induces cell death	[98,99,100,101]
			Syringicin	*Phytophthora syringae*	Induces HR and electrolyte leakage in *N. benthamiana*	[102]
**NLP**	RLP23	BAK1, COI1, HSP90, MEK2, NPR1, SGT1, SOBIR1 and TGA2.2	PcNLP1	*P. cactorum*	Induces cell death	[71]
			Pc11951, Pc107869, Pc109174, Pc118548	*P. capsici*	Induces cell death	[103]
			PcNLP1 to 3, 6 to 10, 13 to 15		Induces cell death	[104]
			PiNPP1.1	*P. infestans*	Induces HR dependent on SGT1 and HSP90	[105]
			PpNLP/NLPPp	*P. parasitica*	Induces cell death	[106,107,108,109,110]
			PsojNIP	*Phytophthora sojae*	Induces cell death dependent on SGT1 and HSP90	[105,111]
			PaNie213/NLPPya	*Phytophthora aphanidermatum*	Induces cell death	[107,108,112]
**CBM**	-	-	CBEL	*P. parasitica*	Induces cell death; activates defense responses via SA, JA and ET signaling pathways	[113,114]
**PL**	-	-	PcPL1, PcPL15, PcPL16, PcPL20	*P. capsici*	Induces cell death	[115]
**GH12**	RXEG1	BAK1, SOBIR1	XEG1	*P. sojae*	Induces cell death; associates with SOBIR1 and BAK1 complex to trigger immune responses	[116,117]
**GH16**	-	-	OPEL	*P. parasitica*	Induces cell death	[118]
**PcF toxin**	-	-	PcF	*P. cactorum*	Induces cell death and PR gene expression in *N. benthamiana*	[119]
			SCR96, SCR99, SCR121		Induces cell death	[120]
			SCR113		Induces cell death	[72]

ND, not determined; NLP, Nep1-like protein; pectate lyase (PL); CBM, carbohydrate binding module; GH, glycoside hydrolase; SAR, systemic acquired resistance.

## References

[1] Kroon L.P.N.M., Brouwer H., De Cock A.W.A.M., Govers F. (2012). The *Phytophthora* genus anno 2012. Phytopathology.

[2] Lamour K.H., Stam R., Jupe J., Huitema E. (2012). The oomycete broad-host-range pathogen *Phytophthora capsici*. Mol. Plant Pathol..

[3] Kamoun S., Furzer O., Jones J.D.G., Judelson H.S., Ali G.S., Dalio R.J.D., Roy S.G., Schena L., Zambounis A., Panabières F. (2015). The Top 10 oomycete pathogens in molecular plant pathology. Mol. Plant Pathol..

[4] Boevink P.C., Birch P.R.J., Turnbull D., Whisson S.C. (2020). Devastating intimacy: The cell biology of plant—*Phytophthora* interactions. New Phytol..

[5] Kinealy C. (2006). This Great Calamity: The Irish Famine 1845-52.

[6] Batini F.E., Hopkins E.R. (1972). *Phytophthora cinnamomi* Rands—a root pathogen of the Jarrah Forest. Aust. For..

[7] Rani G.D. (2008). Advances in Soil Borne Plant Diseases.

[8] Mafurah J.J., Ma H., Zhang M., Xu J., He F., Ye T., Shen D., Chen Y., Rajput N.A., Dou D. (2015). A virulence essential CRN effector of *Phytophthora capsici* suppresses host defense and induces cell death in plant nucleus. PLoS ONE.

[9] Huysmans M., Coll N.S., Nowack M.K. (2017). Dying two deaths—programmed cell death regulation in development and disease. Curr. Opin. Plant Biol..

[10] Tyler B.M., Tripathy S., Zhang X., Dehal P., Jiang R.H.Y., Aerts A., Arredondo F.D., Baxter L., Bensasson D., Beynon J.L. (2006). *Phytophthora* genome sequences uncover evolutionary origins and mechanisms of pathogenesis. Science.

[11] Engelbrecht J., Duong T.A., Prabhu S.A., Seedat M., Berg N.V.D. (2021). Genome of the destructive oomycete *Phytophthora cinnamomi* provides insights into its pathogenicity and adaptive potential. BMC Genom..

[12] Hardham A.R. (2005). *Phytophthora* *cinnamomi*. Mol. Plant Pathol..

[13] Koeck M., Hardham A.R., Dodds P.N. (2011). The role of effectors of biotrophic and hemibiotrophic fungi in infection. Cell. Microbiol..

[14] van Doorn W.G., Beers E.P., Dangl J.L., E Franklin-Tong V., Gallois P., Hara-Nishimura I., Jones A.M., Kawai-Yamada M., Lam E., Mundy J. (2011). Morphological classification of plant cell deaths. Cell Death Differ..

[15] Petrov V., Hille J., Mueller-Roeber B., Gechev T.S. (2015). ROS-mediated abiotic stress-induced programmed cell death in plants. Front. Plant Sci..

[16] Mittler R., Blumwald E. (2010). Genetic engineering for modern agriculture: Challenges and perspectives. Annu. Rev. Plant Biol..

[17] Burke R., Schwarze J., Sherwood O.L., Jnaid Y., McCabe P.F., Kacprzyk J. (2020). Stressed to death: The role of transcription factors in plant programmed cell death induced by abiotic and biotic stimuli. Front. Plant Sci..

[18] Kiraly Z., Barna B., Ersek T. (1972). Hypersensitivity as a consequence, not the cause, of plant resistance to infection. Nature.

[19] Hatsugai N., Kuroyanagi M., Yamada K., Meshi T., Tsuda S., Kondo M., Nishimura M., Hara-Nishimura I. (2004). A plant vacuolar protease, VPE, mediates virus-induced hypersensitive cell death. Science.

[20] Rojo E., Martín R., Carter C., Zouhar J., Pan S., Plotnikova J., Jin H., Paneque M., Serrano J.J.S., Baker B. (2004). VPEγ exhibits a caspase-like activity that contributes to defense against pathogens. Curr. Biol..

[21] Avci U., Petzold H.E., Ismail I.O., Beers E.P., Haigler C.H. (2008). Cysteine proteases XCP1 and XCP2 aid micro-autolysis within the intact central vacuole during xylogenesis in *Arabidopsis* roots. Plant J..

[22] Gao M., Showalter A.M. (1999). Yariv reagent treatment induces programmed cell death in *Arabidopsis* cell cultures and implicates arabinogalactan protein involvement. Plant J..

[23] Mur L.A.J., Kenton P., Lloyd A.J., Ougham H., Prats E. (2008). The hypersensitive response; the centenary is upon us but how much do we know?. J. Exp. Bot..

[24] Zhang M., Li Q., Liu T., Liu L., Shen D., Zhu Y., Liu P., Zhou J.-M., Dou D. (2015). Two cytoplasmic effectors of *Phytophthora sojae* regulate plant cell death via interactions with plant catalases. Plant Physiol..

[25] Daneva A., Gao Z., Van Durme M., Nowack M.K. (2016). Functions and regulation of programmed cell death in plant development. Annu. Rev. Cell Dev. Biol..

[26] Mukhtar M.S., McCormack M.E., Argueso C.T., Pajerowska-Mukhtar K.M. (2016). Pathogen tactics to manipulate plant cell death. Curr. Biol..

[27] Fuchs Y., Steller H. (2011). Programmed cell death in animal development and disease. Cell.

[28] Zhai Z., Ha N., Papagiannouli F., Hamacher-Brady A., Brady N., Sorge S., Bezdan D., Lohmann I. (2012). Antagonistic regulation of apoptosis and differentiation by the Cut transcription factor represents a tumor-suppressing mechanism in *Drosophila*. PLoS Genet..

[29] Aubrey B.J., Kelly G.L., Janic A., Herold M.J., Strasser A. (2018). How does p53 induce apoptosis and how does this relate to p53-mediated tumour suppression?. Cell Death Differ..

[30] Bin Nasir K.H., Takahashi Y., Ito A., Saitoh H., Matsumura H., Kanzaki H., Shimizu T., Ito M., Fujisawa S., Sharma P.C. (2005). High-throughput *in planta* expression screening identifies a class II ethylene-responsive element binding factor-like protein that regulates plant cell death and non-host resistance. Plant J..

[31] Xie H.-T., Wan Z.-Y., Li S., Zhang Y. (2014). Spatiotemporal production of reactive oxygen species by NADPH oxidase is critical for tapetal programmed cell death and pollen development in *Arabidopsis*. Plant Cell.

[32] Lee M.H., Jeon H.S., Kim H.G., Park O.K. (2017). An *Arabidopsis* NAC transcription factor NAC4 promotes pathogen-induced cell death under negative regulation by microRNA164. New Phytol..

[33] Awwad F., Bertrand G., Grandbois M., Beaudoin N. (2019). Auxin protects *Arabidopsis thaliana* cell suspension cultures from programmed cell death induced by the cellulose biosynthesis inhibitors thaxtomin A and isoxaben. BMC Plant Biol..

[34] Yuan X., Wang H., Cai J., Li D., Song F. (2019). NAC transcription factors in plant immunity. Phytopathol. Res..

[35] Kaneda T., Taga Y., Takai R., Iwano M., Matsui H., Takayama S., Isogai A., Che F.-S. (2009). The transcription factor OsNAC4 is a key positive regulator of plant hypersensitive cell death. EMBO J..

[36] Xu X., Chen C., Fan B., Chen Z. (2006). Physical and functional interactions between pathogen-induced *Arabidopsis* WRKY18, WRKY40, and WRKY60 transcription factors. Plant Cell.

[37] Yin L.-L., Xue H.-W. (2012). The MADS29 transcription factor regulates the degradation of the nucellus and the nucellar projection during rice seed development. Plant Cell.

[38] Qi T., Wang J., Huang H., Liu B., Gao H., Liu Y., Song S., Xie D. (2015). Regulation of jasmonate-induced leaf senescence by antagonism between bHLH subgroup IIIe and IIId factors in *Arabidopsis*. Plant Cell.

[39] Ueda H., Kusaba M. (2015). Strigolactone regulates leaf senescence in concert with ethylene in *Arabidopsis*. Plant Physiol..

[40] Glazebrook J. (2005). Contrasting mechanisms of defense against biotrophic and necrotrophic pathogens. Annu. Rev. Phytopathol..

[41] Huang S., Zhang X., Fernando W.G.D. (2020). Directing trophic divergence in plant-pathogen interactions: Antagonistic phytohormones with no doubt?. Front. Plant Sci..

[42] Pieterse C.M.J., Leon-Reyes A., Van der Ent S., Van Wees S.C.M. (2009). Networking by small-molecule hormones in plant immunity. Nat. Chem. Biol..

[43] Birkenbihl R.P., Somssich I.E. (2011). Transcriptional plant responses critical for resistance towards necrotrophic pathogens. Front. Plant Sci..

[44] Kazan K., Lyons R. (2014). Intervention of phytohormone pathways by pathogen effectors. Plant Cell.

[45] Van Durme M., Nowack M.K. (2016). Mechanisms of developmentally controlled cell death in plants. Curr. Opin. Plant Biol..

[46] Wilkins K.A., Bosch M., Haque T., Teng N., Poulter N.S., Franklin-Tong V.E. (2015). Self-incompatibility-induced programmed cell death in field poppy pollen involves dramatic acidification of the incompatible pollen tube cytosol. Plant Physiol..

[47] Coll N.S., Epple P., Dangl J.L. (2011). Programmed cell death in the plant immune system. Cell Death Differ..

[48] Herrera-Vãsquez A., Salinas P., Holuigue L. (2015). Salicylic acid and reactive oxygen species interplay in the transcriptional control of defense genes expression. Front. Plant Sci..

[49] Lorang J., Kidarsa T., Bradford C.S., Gilbert B., Curtis M., Tzeng S.-C., Maier C.S., Wolpert T.J. (2012). Tricking the guard: Exploiting plant defense for disease susceptibility. Science.

[50] Lorang J. (2019). Necrotrophic exploitation and subversion of plant defense: A lifestyle or just a phase, and implications in breeding resistance. Phytopathology.

[51] Stam R., Jupe J., Howden A.J.M., Morris J.A., Boevink P.C., Hedley P.E., Huitema E. (2013). Identification and characterisation CRN effectors in *Phytophthora capsici* shows modularity and functional diversity. PLoS ONE.

[52] Münch S., Lingner U., Floss D.S., Ludwig N., Sauer N., Deising H.B. (2008). The hemibiotrophic lifestyle of *Colletotrichum* species. J. Plant Physiol..

[53] Jupe J., Stam R., Howden A.J., Morris J.A., Zhang R., Hedley P.E., Huitema E. (2013). *Phytophthora capsici*-tomato interaction features dramatic shifts in gene expression associated with a hemi-biotrophic lifestyle. Genome Biol..

[54] Liu T., Ye W., Ru Y., Yang X., Gu B., Tao K., Lu S., Dong S., Zheng X., Shan W. (2011). Two host cytoplasmic effectors are required for pathogenesis of *Phytophthora sojae* by suppression of host defenses. Plant Physiol..

[55] Maximo H.J., Dalio R.O., Litholdo C.G., Felizatti H.L., Machado M.A. (2019). PpCRN7 and PpCRN20 of *Phythophthora parasitica* regulate plant cell death leading to enhancement of host susceptibility. BMC Plant Biol..

[56] Wang S., Welsh L., Thorpe P., Whisson S.C., Boevink P.C., Birch P.R.J. (2018). The *Phytophthora infestans* haustorium is a site for secretion of diverse classes of infection-associated proteins. MBio.

[57] Judelson H.S., Ah-Fong A.M.V. (2019). Exchanges at the plant-oomycete interface that influence disease. Plant Physiol..

[58] Dong S., Kong G., Qutob D., Yu X., Tang J., Kang J., Dai T., Wang H., Gijzen M., Wang Y. (2012). The NLP toxin family in *Phytophthora sojae* includes rapidly evolving groups that lack necrosis-inducing activity. Mol. Plant Microbe Interact..

[59] Ah-Fong A.M.V., Shrivastava J., Judelson H.S. (2017). Lifestyle, gene gain and loss, and transcriptional remodeling cause divergence in the transcriptomes of *Phytophthora infestans* and *Pythium ultimum* during potato tuber colonization. BMC Genom..

[60] Van Damme M., Bozkurt T.O., Cakir C., Schornack S., Sklenář J., Jones A.M.E., Kamoun S. (2012). The Irish potato famine pathogen *Phytophthora infestans* translocates the CRN8 kinase into host plant cells. PLoS Pathog..

[61] Li Q., Ai G., Shen D., Zou F., Wang J., Bai T., Chen Y., Li S., Zhang M., Jing M. (2019). A *Phytophthora capsici* effector targets ACD11 binding partners that regulate ROS-mediated defense response in Arabidopsis. Mol. Plant.

[62] Toljamo A., Blande D., Munawar M., Kärenlampi S.O., Kokko H. (2019). Expression of the GAF sensor, carbohydrate-active Enzymes, elicitins, and RXLRs differs markedly between two *Phytophthora cactorum* isolates. Phytopathology.

[63] Hardham A.R., Blackman L.M. (2018). *Phytophthora* *cinnamomi*. Mol. Plant Pathol..

[64] Kamoun S. (2006). A catalogue of the effector secretome of plant pathogenic oomycetes. Annu. Rev. Phytopathol..

[65] Gijzen M., Nürnberger T. (2006). Nep1-like proteins from plant pathogens: Recruitment and diversification of the NPP1 domain across taxa. Phytochemistry.

[66] Lamour K., Kamoun S. (2009). Oomycete Genetics and Genomics: Diversity, Interactions and Research Tools.

[67] Wang Y., Hu D., Zhang Z., Ma Z., Zheng X., Li D. (2003). Purification and immune cytolocalization of a novel *Phytophthora boehmeriae* protein inducing the hypersensitive response and systemic acquired resistance in tobacco and Chinese cabbage. Physiol. Mol. Plant Pathol..

[68] Zhang Z.-G., Wang Y.-C., Li J., Ji R., Shen G., Wang S.-C., Zhou X., Zheng X.-B. (2004). The role of SA in the hypersensitive response and systemic acquired resistance induced by elicitor PB90 from *Phytophthora boehmeriae*. Physiol. Mol. Plant Pathol..

[69] Chen Q., Chen Z., Lu L., Jin H., Sun L., Yu Q., Xu H., Yang F., Fu M., Li S. (2013). Interaction between abscisic acid and nitric oxide in PB90-induced catharanthine biosynthesis of *Catharanthus roseus* cell suspension cultures. Biotechnol. Prog..

[70] Huet J.-C., Pernollet M.M.J.-C. (1993). Amino acid sequence of the α-elicitin secreted by *Phytophthora cactorum*. Phytochemistry.

[71] Chen X.-R., Zhang B.-Y., Xing Y.-P., Li Q.-Y., Li Y.-P., Tong Y.-H., Xu J.-Y. (2014). Transcriptomic analysis of the phytopathogenic oomycete *Phytophthora cactorum* provides insights into infection-related effectors. BMC Genom..

[72] Chen X.R., Huang S.X., Zhang Y., Sheng G.L., Zhang B.Y., Li Q.Y., Zhu F., Xu J.Y. (2017). Transcription profiling and identification of infection-related genes in *Phytophthora cactorum*. Mol. Genet. Genom..

[73] Ricci P., Bonnet P., Huet J.-C., Sallantin M., Beauvais-Cante F., Bruneteau M., Billard V., Michel G., Pernollet J.-C. (1989). Structure and activity of proteins from pathogenic fungi *Phytophthora* eliciting necrosis and acquired resistance in tobacco. Eur. J. Biochem..

[74] Liu Z.-Q., Qiu A.-L., Shi L.-P., Cai J.-S., Huang X.-Y., Yang S., Wang B., Shen L., Huang M.-K., Mou S.-L. (2015). SRC2-1 is required in PcINF1-induced pepper immunity by acting as an interacting partner of PcINF1. J. Exp. Bot..

[75] Liu Z.-Q., Liu Y.-Y., Shi L.-P., Yang S., Shen L., Yu H.-X., Wang R.-Z., Wen J.-Y., Tang Q., Hussain A. (2016). SGT1 is required in PcINF1/SRC2-1 induced pepper defense response by interacting with SRC2-1. Sci. Rep..

[76] Billard V., Bruneteau M., Bonnet P., Ricci P., Pernollet J., Huet J., Vergne A., Richard G., Michel G. (1988). Chromatographic purification and characterization of elicitors of necrosis on tobacco produced by incompatible *Phytophthora species*. J. Chromatogr..

[77] Huet J.-C., Pernollet J.-C. (1989). Amino acid sequence of cinnamomin, a new member of the elicitin family, and its comparison to cryptogein and capsicein. FEBS Lett..

[78] Mishra A.K., Sharma K., Misra R.S. (2009). Purification and characterization of elicitor protein from *Phytophthora colocasiae* and basic resistance in *Colocasia esculenta*. Microbiol. Res..

[79] Galiana E., Bonnet P., Conrod S., Keller H., Panabieres F., Ponchet M., Poupet A., Ricci P. (1997). RNase activity prevents the growth of a fungal pathogen in tobacco leaves and increases upon induction of systemic acquired resistance with elicitin. Plant Physiol..

[80] Mikes V., Milat M.-L., Ponchet M., Ricci P., Blein J.-P. (1997). The fungal elicitor cryptogein is a sterol carrier protein. FEBS Lett..

[81] Leborgne-Castel N., Lherminier J., Der C., Fromentin J., Houot V., Simon-Plas F. (2008). The plant defense elicitor cryptogein stimulates clathrin mediated endocytosis correlated with reactive oxygen species production in bright yellow-2 tobacco cells. Plant Physiol..

[82] Coursol S., Fromentin J., Noirot E., Brière C., Robert F., Morel J., Liang Y., Lherminier J., Simon-Plas F. (2015). Long-chain bases and their phosphorylated derivatives differentially regulate cryptogein-induced production of reactive oxygen species in tobacco (*Nicotiana tabacum*) BY-2 cells. New Phytol..

[83] Kulik A., Noirot E., Grandperret V., Bourque S., Fromentin J., Salloignon P., Truntzer C., Dobrowolska G., Simon-Plas F., Wendehenne D. (2015). Interplays between nitric oxide and reactive oxygen species in cryptogein signalling. Plant Cell Environ..

[84] Ptáčková N., Klempová J., Obořil M., Nedělová S., Lochman J., Kašparovský T. (2015). The effect of cryptogein with changed abilities to transfer sterols and altered charge distribution on extracellular alkalinization, ROS and NO generation, lipid peroxidation and LOX gene transcription in *Nicotiana tabacum*. Plant Physiol. Biochem..

[85] Starý T., Satková P., Piterková J., Mieslerová B., Luhová L., Mikulík J., Kašparovský T., Petřivalský M., Lochman J. (2019). The elicitin β-cryptogein’s activity in tomato is mediated by jasmonic acid and ethylene signalling pathways independently of elicitin-sterol interactions. Planta.

[86] Huet J.-C., Nespoulous C., Pernollet J.-C. (1992). Structures of elicitin isoforms secreted by *Phytophthora drechsleri*. Phytochemistry.

[87] Capasso R., Di Maro A., Cristinzio G., De Martino A., Chambery A., Daniele A., Sannino F., Testa A., Parente A. (2008). Isolation, characterization and structure-elicitor activity relationships of hibernalin and its two oxidized forms from *Phytophthora hibernalis*. Carne 1925. J. Biochem..

[88] Huet J., Sallé-Tourne M., Pernollet J. (1994). Amino acid sequence and toxicity of the alpha elicitin secreted with ubiquitin by *Phytophthora infestans*. Mol. Plant Microbe Interact..

[89] Kamoun S., van West P., de Jong A.J., de Groot K.E., Vleeshouwers V.G.A.A., Govers F. (1997). A gene encoding a protein elicitor of *Phytophthora infestans* is down-regulated during infection of potato. Mol. Plant Microbe Interact..

[90] Kamoun S., Van West P., Vleeshouwers V.G.A.A., De Groot K.E., Govers F. (1998). Resistance of *Nicotiana benthamiana* to *Phytophthora infestans* is mediated by the recognition of the elicitor protein INF1. Plant Cell.

[91] Kanzaki H., Saitoh H., Ito A., Fujisawa S., Kamoun S., Katou S., Yoshioka H., Terauchi R. (2003). Cytosolic HSP90 and HSP70 are essential components of INF1-mediated hypersensitive response and non-host resistance to *Pseudomonas cichorii* in *Nicotiana benthamiana*. Mol. Plant Pathol..

[92] Huitema E., Vleeshouwers V.G., Cakir C., Kamoun S., Govers F. (2005). Differences in intensity and specificity of hypersensitive response induction in *Nicotiana* spp. by INF1, INF2A, and INF2B of *Phytophthora infestans*. Mol. Plant Microbe Interact..

[93] Du J., Verzaux E., Chaparro-Garcia A., Bijsterbosch G., Keizer L.C.P., Zhou J., Liebrand T.W.H., Xie C., Govers F., Robatzek S. (2015). Elicitin recognition confers enhanced resistance to *Phytophthora infestans* in potato. Nat. Plants.

[94] Huet J.-C., Pernollet J.-C. (1993). Sequences of acidic and basic elicitin isoforms secreted by *Phytophthora megasperma*. Phytochemistry.

[95] Baillieul F., de Ruffray P., Kauffmann S. (2003). Molecular cloning and biological activity of α-, β-, and γ-megaspermin, three elicitins secreted by *Phytophthora megasperma* H20. Plant Physiol..

[96] Baillieul F., Genetet I., Kopp M., Saindrenan P., Fritig B., Kauffmann S. (1995). A new elicitor of the hypersensitive response in tobacco: A fungal glycoprotein elicits cell death, expression of defence genes, production of salicylic acid, and induction of systemic acquired resistance. Plant J..

[97] Churngchow N., Rattarasarn M. (2000). The elicitin secreted by *Phytophthora palmivora*, a rubber tree pathogen. Phytochemistry.

[98] Nespoulous C., Huet J.-C., Pernollet J.-C. (1992). Structure-function relationships of α and β elicitins, signal proteins involved in the plant-*Phytophthora* interaction. Planta.

[99] Kamoun S., Klucher K.M., Coffey M.D., Tyler B.M. (1993). A gene encoding a host-specific elicitor protein of *Phytophthora parasitica*. Mol. Plant Microbe Interact..

[100] Mouton-Perronnet F., Bruneteau M., Denoroy L., Bouliteau P., Ricci P., Bonnet P., Michel G. (1995). Elicitin produced by an isolate of *Phytophthora parasitica* pathogenic to tobacco. Phytochemistry.

[101] Capasso R., Cristinzio G., Evidente A., Visca C., Ferranti P., Blanco F.D.V., Parente A. (1999). Elicitin 172 from an isolate of *Phytophthora nicotianae* pathogenic to tomato. Phytochemistry.

[102] Capasso R., Cristinzio G., Di Maro A., Ferranti P., Parente A. (2001). Syringicin, a new α-elicitin from an isolate of *Phytophthora syringae*, pathogenic to citrus fruit. Phytochemistry.

[103] Chen X.-R., Huang S.-X., Zhang Y., Sheng G.-L., Li Y.-P., Zhu F. (2018). Identification and functional analysis of the NLP-encoding genes from the phytopathogenic oomycete *Phytophthora capsici*. Mol. Genet. Genom..

[104] Feng B.-Z., Zhu X.-P., Fu L., Lv R.-F., Storey D., Tooley P., Zhang X.-G. (2014). Characterization of necrosis-inducing NLP proteins in *Phytophthora capsici*. BMC Plant Biol..

[105] Kanneganti T.-D., Huitema E., Cakir C., Kamoun S. (2006). Synergistic interactions of the plant cell death pathways induced by *Phytophthora infestans* Nep1-like protein PiNPP1.1 and INF1 elicitin. Mol. Plant Microbe Interact..

[106] Fellbrich G., Romanski A., Varet A., Blume B., Brunner F., Engelhardt S., Felix G., Kemmerling B., Krzymowska M., Nürnberger T. (2002). NPP1, a *Phytophthora*-associated trigger of plant defense in parsley and *Arabidopsis*. Plant J..

[107] Qutob D., Kemmerling B., Brunner F., Kufner I., Engelhardt S., Gust A.A., Luberacki B., Seitz H.U., Stahl D., Rauhut T. (2006). Phytotoxicity and innate immune responses induced by Nep1-like proteins. Plant Cell.

[108] Ottmann C., Luberacki B., Küfner I., Koch W., Brunner F., Weyand M., Mattinen L., Pirhonen M., Anderluh G., Seitz H.U. (2009). A common toxin fold mediates microbial attack and plant defense. Proc. Natl. Acad. Sci. USA.

[109] Böhm H., Albert I., Oome S., Raaymakers T.M., Van Den Ackerveken G., Nürnberger T. (2014). A conserved peptide pattern from a widespread microbial virulence factor triggers pattern-induced immunity in *Arabidopsis*. PLoS Pathog..

[110] Albert I., Böhm H., Albert M., Feiler C.E., Imkampe J., Wallmeroth N., Brancato C., Raaymakers T.M., Oome S., Zhang H. (2015). An RLP23-SOBIR1-BAK1 complex mediates NLP triggered immunity. Nat. Plants..

[111] Qutob D., Kamoun S., Gijzen M. (2002). Expression of a *Phytophthora sojae* necrosis-inducing protein occurs during transition from biotrophy to necrotrophy. Plant J..

[112] Veit S., Wörle J.M., Nürnberger T., Koch W., Seitz H.U. (2001). A novel protein elicitor (PaNie) from *Pythium aphanidermatum* induces multiple defense responses in carrot, *Arabidopsis*, and tobacco. Plant Physiol..

[113] Mateos F.V., Rickauer M., Esquerré-Tugayé M.-T. (1997). Cloning and characterization of a cDNA encoding an elicitor of *Phytophthora parasitica var. nicotianae* that shows cellulose-binding and lectin-like activities. Mol. Plant Microbe Interact..

[114] Khatib M., Lafitte C., Esquerré-Tugayé M., Bottin A., Rickauer M. (2004). The CBEL elicitor of *Phytophthora parasitica var. nicotianae* activates defence in *Arabidopsis thaliana* via three different signalling pathways. New Phytol..

[115] Fu L., Zhu C., Ding X., Yang X., Morris P.F., Tyler B.M., Zhang X. (2015). Characterization of cell-death-inducing members of the pectate lyase gene family in *Phytophthora capsici* and their contributions to infection of pepper. Mol. Plant Microbe Interact..

[116] Ma Z., Song T., Zhu L., Ye W., Wang Y., Shao Y., Dong S., Zhang Z., Dou D., Zheng X. (2015). A *Phytophthora sojae* glycoside hydrolase 12 protein is a major virulence factor during soybean infection and is recognized as a PAMP. Plant Cell.

[117] Wang Y., Xu Y., Sun Y., Wang H., Qi J., Wan B., Ye W., Lin Y., Shao Y., Dong S. (2018). Leucine rich repeat receptor-like gene screen reveals that *Nicotiana* RXEG1 regulates glycoside hydrolase 12 MAMP detection. Nat. Commun..

[118] Chang Y.-H., Yan H.-Z., Liou R.-F. (2015). A novel elicitor protein from *Phytophthora parasitica* induces plant basal immunity and systemic acquired resistance. Mol. Plant Pathol..

[119] Orsomando G., Lorenzi M., Raffaelli N., Rizza M.D., Mezzetti B., Ruggieri S. (2001). Phytotoxic protein PcF, purification, characterization, and cDNA sequencing of a novel hydroxyproline-containing factor secreted by the strawberry pathogen *Phytophthora cactorum*. J. Biol. Chem..

[120] Chen X.-R., Li Y.-P., Li Q.-Y., Xing Y.-P., Liu B.-B., Tong Y.-H., Xu J.-Y. (2016). SCR96, a small cysteine-rich secretory protein of *Phytophthora cactorum*, can trigger cell death in the *Solanaceae* and is important for pathogenicity and oxidative stress tolerance. Mol. Plant Pathol..

[121] Duclos J., Fauconnier A., Coelho A.-C., Bollen A., Cravador A., Godfroid E. (1998). Identification of an elicitin gene cluster in *Phytophthora cinnamomi*. DNA Seq..

[122] Jiang R.H.Y., Tyler B.M., Whisson S.C., Hardham A.R., Govers F. (2006). Ancient origin of elicitin gene clusters in *Phytophthora* genomes. Mol. Biol. Evol..

[123] Osman H., Vauthrin S., Mikes V., Milat M.-L., Panabières F., Marais A., Brunie S., Maume B., Ponchet M., Blein J.-P. (2001). Mediation of elicitin activity on tobacco is assumed by elicitin-sterol complexes. Mol. Biol. Cell.

[124] Rodrigues M.L., Archer M., Martel P., Miranda S., Thomaz M., Enguita F.J., Baptista R.P., e Melo E.P., Sousa N., Cravador A. (2006). Crystal structures of the free and sterol-bound forms of β-cinnamomin. Biochim. Biophys. Acta Proteins Proteom..

[125] Boissy G., de La Fortelle E., Kahn R., Huet J.-C., Bricogne G., Pernollet J.-C., Brunie S. (1996). Crystal structure of a fungal elicitor secreted by *Phytophthora cryptogea*, a member of a novel class of plant necrotic proteins. Structure.

[126] Derevnina L., Dagdas Y.F., De la Concepcion J.C., Białas A., Kellner R., Petre B., Domazakis E., Du J., Wu C.-H., Lin X. (2016). Nine things to know about elicitins. New Phytol..

[127] Nie J., Yin Z., Li Z., Wu Y., Huang L. (2019). A small cysteine-rich protein from two kingdoms of microbes is recognized as a novel pathogen-associated molecular pattern. New Phytol..

[128] Dokládal L., Obořil M., Stejskal K., Zdráhal Z., Ptáčková N., Chaloupková R., Damborský J., Kašparovský T., Jeandroz S., Žd’árská M. (2012). Physiological and proteomic approaches to evaluate the role of sterol binding in elicitin-induced resistance. J. Exp. Bot..

[129] Adachi H., Nakano T., Miyagawa N., Ishihama N., Yoshioka M., Katou Y., Yaeno T., Shirasu K., Yoshioka H. (2015). WRKY transcription factors phosphorylated by MAPK regulate a plant immune NADPH oxidase in *Nicotiana benthamiana*. Plant Cell.

[130] Bos J.I.B., Kanneganti T.-D., Young C., Cakir C., Huitema E., Win J., Armstrong M.R., Birch P.R.J., Kamoun S. (2006). The C-terminal half of *Phytophthora infestans* RXLR effector AVR3a is sufficient to trigger R3a-mediated hypersensitivity and suppress INF1-induced cell death in *Nicotiana benthamiana*. Plant J..

[131] Haas B.J., Kamoun S., Zody M.C., Jiang R.H.Y., Handsaker R.E., Cano L.M., Grabherr M., Kodira C.D., Raffaele S., Torto-Alalibo T. (2009). Genome sequence and analysis of the Irish potato famine pathogen *Phytophthora infestans*. Nature.

[132] Masago H. (1977). Selective inhibition of *Pythium* spp. on a medium for direct isolation of *Phytophthora* spp. from soils and plants. Phytopathology.

[133] Cabral A., Oome S., Sander N., Küfner I., Nürnberger T., Van den Ackerveken G. (2012). Nontoxic Nep1-like proteins of the downy mildew pathogen *Hyaloperonospora arabidopsidis*: Repression of necrosis-inducing activity by a surface-exposed region. Mol. Plant Microbe Interact..

[134] Oome S., Van den Ackerveken G. (2014). Comparative and functional analysis of the widely occurring family of Nep1-like proteins. Mol. Plant Microbe Interact..

[135] Lenarčič T., Albert I., Böhm H., Hodnik V., Pirc K., Zavec A.B., Podobnik M., Pahovnik D., Žagar E., Pruitt R. (2017). Eudicot plant-specific sphingolipids determine host selectivity of microbial NLP cytolysins. Science.

[136] Lenarčič T., Pric K., Hodnik V., Albert I., Borišek J., Magistrato A., Nürnberger T., Podobnik M., Anderluh G. (2019). Molecular basis for functional diversity among microbial Nep1-like proteins. PLoS Pathog..

[137] Bae H., Bowers J.H., Tooley P.W., Bailey B.A. (2005). NEP1 orthologs encoding necrosis and ethylene inducing proteins exist as a multigene family in *Phytophthora megakarya*, causal agent of black pod disease on cacao. Mycol. Res..

[138] Chen X.-R., Xing Y.-P., Li Y.-P., Tong Y.-H., Xu J.-Y. (2013). RNA-Seq reveals infection-related gene expression changes in *Phytophthora capsici*. PLoS ONE.

[139] Martins I.M., Meirinho S., Costa R., Cravador A., Choupina A. (2019). Cloning, characterization, in vitro and *in planta* expression of a necrosis-inducing *Phytophthora* protein 1 gene npp1 from *Phytophthora cinnamomi*. Mol. Biol. Rep..

[140] Win J., Morgan W., Bos J., Krasileva K., Cano L.M., Chaparro-Garcia A., Ammar R., Staskawicz B.J., Kamoun S. (2007). Adaptive evolution has targeted the C-terminal domain of the RXLR effectors of plant pathogenic oomycetes. Plant Cell.

[141] Dou D., Kale S.D., Wang X., Jiang R.H., Bruce N.A., Arredondo F.D., Zhang X., Tyler B.M. (2008). RXLR-mediated entry of *Phytophthora sojae* effector Avr1b into soybean cells does not require pathogen-encoded machinery. Plant Cell.

[142] Kale S.D., Gu B., Capelluto D.G., Dou D., Feldman E., Rumore A., Arredondo F.D., Hanlon R., Fudal I., Rouxel T. (2010). External lipid PI3P mediates entry of eukaryotic pathogen effectors into plant and animal host cells. Cell.

[143] Joubert M., Backer R., Engelbrecht J., Berg N.V.D. (2021). Expression of several *Phytophthora cinnamomi* putative RxLRs provides evidence for virulence roles in avocado. PLoS ONE.

[144] Wang S., McLellan H., Bukharova T., He Q., Murphy F., Shi J., Sun S., van Weymers P., Ren Y., Thilliez G. (2019). *Phytophthora infestans* RXLR effectors act in concert at diverse subcellular locations to enhance host colonization. J. Exp. Bot..

[145] Yang B., Wang Q., Jing M., Guo B., Wu J., Wang H., Wang Y., Lin L., Ye W., Dong S. (2017). Distinct regions of the *Phytophthora* essential effector Avh238 determine its function in cell death activation and plant immunity suppression. New Phytol..

[146] Li H., Wang H., Jing M., Zhu J., Guo B., Wang Y., Lin Y., Chen H., Kong L., Ma Z. (2018). A *Phytophthora* effector recruits a host cytoplasmic transacetylase into nuclear speckles to enhance plant susceptibility. eLife.

[147] Chen X.-R., Zhang Y., Li H.-Y., Zhang Z.-H., Sheng G.-L., Li Y.-P., Xing Y.-P., Huang S.-X., Tao H., Kuan T. (2019). The RXLR effector PcAvh1 is required for full virulence of *Phytophthora capsici*. Mol. Plant Microbe Interact..

[148] Branco I., Choupina A. (2020). In Silico characterization of the Phytopathogenic efector, Avr3a, from *Phytophthora cinnamomi*. J. Basic Appl. Sci..

[149] Torto T.A., Li S., Styler A., Huitema E., Testa A., Gow N.A.R., van West P., Kamoun S. (2003). EST mining and functional expression assays identify extracellular effector proteins from the plant pathogen *Phytophthora*. Genome Res..

[150] Schornack S., van Damme M., Bozkurt T.O., Cano L.M., Smoker M., Thines M., Gaulin E., Kamoun S., Huitema E. (2010). Ancient class of translocated oomycete effectors targets the host nucleus. Proc. Natl. Acad. Sci. USA.

[151] Stam R., Howden A.J.M., Delgado-Cerezo M., Amaro T.M.M.M., Motion G.B., Pham J., Huitema E. (2013). Characterization of cell death inducing *Phytophthora capsici* CRN effectors suggests diverse activities in the host nucleus. Front. Plant Sci..

[152] Amaro T.M.M.M., Thilliez G.J.A., Motion G.B., Huitema E. (2017). A perspective on CRN Proteins in the genomics age: Evolution, classification, delivery and function revisited. Front. Plant Sci..

[153] Cheung F., Win J., Lang J.M., Hamilton J., Vuong H., Leach J.E., Kamoun S., Lévesque C.A., Tisserat N., Buell C.R. (2008). Analysis of the *Pythium ultimum* transcriptome using Sanger and pyrosequencing approaches. BMC Genom..

[154] Gaulin E., Madoui M.-A., Bottin A., Jacquet C., Mathé C., Couloux A., Wincker P., Dumas B. (2008). Transcriptome of *Aphanomyces euteiches*: New oomycete putative pathogenicity factors and metabolic pathways. PLoS ONE.

[155] Lévesque C.A., Brouwer H., Cano L., Hamilton J.P., Holt C., Huitrma E., Raffaele S., Robideau G.P., Thines M., Win J. (2010). Genome sequence of the necrotrophic plant pathogen *Pythium ultimum* reveals original pathogenicity mechanisms and effector repertoire. Genome Biol..

[156] Meyer F.E., Shuey L.S., Naidoo S., Mamni T., Berger D.K., Myburg A.A., Berg N.V.D., Naidoo S. (2016). Dual RNA-sequencing of *Eucalyptus nitens* during *Phytophthora cinnamomi* challenge reveals pathogen and host factors influencing compatibility. Front. Plant Sci..

[157] Zhang D., Burroughs A.M., Vidal N.D., Iyer L.M., Aravind L. (2016). Transposons to toxins: The provenance, architecture and diversification of a widespread class of eukaryotic effectors. Nucleic Acids Res..

[158] Li Q., Zhang M., Shen D., Liu T., Chen Y., Zhou J.M., Dou D. (2016). A *Phytophthora sojae* effector PsCRN63 forms homo-/hetero-dimers to suppress plant immunity via an inverted association manner. Sci. Rep..

[159] Bendahmane A., Querci M., Kanyuka K., Baulcombe D.C. (2000). Agrobacterium transient expression system as a tool for the isolation of disease resistance genes: Application to the Rx2 locus in potato. Plant J..

[160] Van der Hoorn R.A.L., Laurent F., Roth R., De Wit P.J.G.M. (2000). Agroinfiltration is a versatile tool that facilitates comparative analyses of Avr 9/Cf-9-induced and Avr4/Cf-4-induced necrosis. Mol. Plant Microbe Interact..

[161] Wroblewski T., Tomczak A., Michelmore R. (2005). Optimization of Agrobacterium-mediated transient assays of gene expression in lettuce, tomato and *Arabidopsis*. Plant Biotechnol. J..

[162] Lee M.W., Yang Y. (2006). Transient expression assay by agroinfiltration of leaves. Arabidopsis Protocols.

[163] Ramirez-Garcés D., Camborde L., Pel M.J.C., Jauneau A., Martinez Y., Néant I., Leclerc C., Moreau M., Dumas B., Gaulin E. (2016). CRN 13 candidate effectors from plant and animal eukaryotic pathogens are DNA-binding proteins which trigger host DNA damage response. New Phytol..

[164] Goodin M.M., Zaitlin D., Naidu R.A., Lommel S.A. (2008). *Nicotiana benthamiana*: Its history and future as a model for plant–pathogen interactions. Mol. Plant Microbe Interact..

[165] Kamoun S., Goodwin S.B. (2007). Fungal and oomycete genes galore. New Phytol..

[166] Studholme D., McDougal R., Sambles C., Hansen E., Hardy G., Grant M., Ganley R.J., Williams N.M. (2016). Genome sequences of six *Phytophthora* species associated with forests in New Zealand. Genom. Data.

[167] Langmuir A.L., Beech P.L., Richardson M.F. (2017). Draft genomes of two Australian strains of the plant pathogen, *Phytophthora cinnamomi*. F1000Research.

[168] Kunjeti S.G., Evans T.A., Marsh A.G., Gregory N.F., Kunjeti S., Meyers B.C., Kalavacharla V.S., Donofrio N.M. (2012). RNA-Seq reveals infection-related global gene changes in *Phytophthora phaseoli*, the causal agent of lima bean downy mildew. Mol. Plant Pathol..

[169] Westermann A.J., Gorski S.A., Vogel J. (2012). Dual RNA-seq of pathogen and host. Nat. Rev. Microbiol..

[170] Hayden K.J., Garbelotto J.M., Knaus B.J., Cronn R.C., Wright J.W. (2014). Dual RNA-seq of the plant pathogen *Phytophthora ramorum* and its tanoak host. Tree Genet. Genomes.

[171] Reitmann A., Berger D.K., Berg N.V.D. (2017). Putative pathogenicity genes of *Phytophthora cinnamomi* identified via RNA-Seq analysis of pre-infection structures. Eur. J. Plant Pathol..

[172] Judelson H.S. (1996). Recent advances in the genetics of oomycete plant pathogens. Mol. Plant Microbe Interact..

[173] Dai T., Xu Y., Yang X., Jiao B., Qiu M., Xue J., Arredondo F., Tyler B.M. (2021). An improved transformation system for *Phytophthora cinnamomi* using green fluorescent protein. Front. Microbiol..

[174] Bailey A.M., Mena G.L., Herrera-Estrella L.R. (1991). Genetic transformation of the plant pathogens *Phytophthora capsici* and *Phytophthora parasitica*. Nucleic Acids Res..

[175] Judelson H.S., Tyler B.M., Michelmore R.W. (1991). Transformation of the oomycete pathogen, *Phytophthora infestans*. Mol. Plant-Microbe Interact..

